# Spontaneous pregnancy after fertility‐sparing surgery and adjuvant chemotherapy for advanced pure dysgerminoma: A case report

**DOI:** 10.1002/ccr3.9020

**Published:** 2024-05-30

**Authors:** Göksu Göç, Alida Göç, Gezim Kastrati, Dardan Baftiu, Fisnik Kurshumliu

**Affiliations:** ^1^ Department of Obstetrics and Gynaecology American Hospital Prishtina Kosovo; ^2^ Institute of Anatomical Pathology, School of Medicine University Prishtina Prishtina Kosovo

**Keywords:** adjuvant chemotherapy, dysgerminoma, fertility‐sparing surgery, germ cell neoplasms, ovary, pregnancy

## Abstract

**Key Clinical Message:**

Fertility‐sparing surgery and appropriate adjuvant chemotherapy for advanced malignant ovarian germ cell tumors have excellent survival results and promising reproductive and obstetric outcomes.

**Abstract:**

This case report aims to demonstrate the potential feasibility and success of fertility‐sparing surgery (FSS) coupled with adjuvant chemotherapy in treating advanced malignant ovarian germ cell tumor (MOGCT), focusing on pure dysgerminoma, fertility, and achieving spontaneous pregnancy. The patient was a 23‐year‐old female who initially presented with complaints of abdominal distension and a palpable mass and was subsequently diagnosed with advanced MOGCT. The patient provided a complete clinical and radiological response to FSS with complete surgical staging and cisplatin‐based chemotherapy (bleomycin, etoposide, and cisplatin). Despite being diagnosed with advanced MOGCT and treated with FSS and adjuvant chemotherapy, she later experienced spontaneous pregnancy, giving birth to a healthy child. This case study demonstrated the potential for successful fertility preservation and pregnancy in advanced‐stage MOGCT patients treated with personalized treatment approaches. Nevertheless, a broader investigation is needed to understand the relevant complex dynamics and to ascertain whether FSS with adjuvant chemotherapy could be a reliable approach in treating advanced MOGCT.

## Graphical abstract

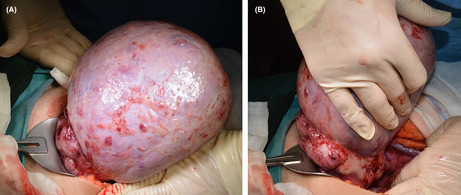

## 1 INTRODUCTION

Malignant ovarian germ cell tumor (MOGCT), a rare form of ovarian malignancy, predominantly affects adolescents and young women of reproductive age.^1^, ^2^ According to the literature, 193 patients have been diagnosed with gestational non‐epithelial ovarian cancer, and treated with chemotherapy during pregnancy. Among them, 145 were cases of MOGCTs.^3^ This group of ovarian cancers includes various subtypes, including dysgerminoma, yolk sac tumors, embryonal carcinoma, non‐gestational choriocarcinoma, mixed germ cell tumors, and immature teratomas, each with distinct characteristics.^4^, ^5^, ^6^ Let‐7, which is likely to be involved in the pathogenesis of these tumors, is a group of nine miRNAs that function as important tumor suppressor genes. Let‐7 is negatively regulated by the RNA‐binding protein LIN‐28 homolog A (LIN28), which controls the pluripotency of embryonic stem cells. Importantly, LIN28‐positive germ cell tumors have been shown to have reduced levels of let‐7 miRNA, therefore suggesting that the LIN28/let‐7 pathway could have a significant role in the pathogenesis of germ cell tumors.^7^ Dysgerminoma, akin to male seminoma, represents the most prevalent histological variant and immature teratoma and is associated with relatively high bilaterality rates.^5^, ^8^, ^9^

Platinum‐based chemotherapy regimens have proven effective in extending survival and preserving fertility.^1^, ^10^ Given the chemosensitivity of these tumor cells, fertility‐sparing surgery (FSS) has become a preferred treatment approach, particularly in patients desiring to preserve their reproductive capability. FSS, which involves complete staging and the preservation of at least the uterine corpus and a portion of one ovary, has emerged as the primary treatment modality in patients with early‐stage MOGCT.^10^, ^11^, ^12^ However, a couple of studies in which the potential risks of FSS use have been extensively discussed have stated that FSS use can be justifiable in advanced‐stage MOGCT patients.^12^, ^13^

Menstrual and reproductive outcomes in patients who survived MOGCT are reportedly similar to those of age‐matched healthy women.^1^, ^14^ Ovarian function is typically restored following three or four cycles of platinum‐based therapy.^14^ However, fertility rates vary significantly, including among patients with advanced MOGCT.^3^, ^10^, ^13^

In this context, in this case study, an advanced MOGCT patient who underwent complete staging and was treated with FSS coupled with adjuvant cisplatin‐based chemotherapy and still achieved spontaneous pregnancy is presented.

## 2 CASE PRESENTATION

### 2.1 Case history and examination

A 23‐year‐old woman presented with a 6‐month history of abdominal distention and a progressive increasing palpable mass in the lower abdominal quadrants. At the time of admission, the size of the mass was equivalent to the swelling in a six‐month‐pregnant woman. Her personal and familial medical history was unremarkable.

Her physical examination revealed a firm, smooth, and painless palpable mass in the right lower quadrant.

### 2.2 Methods

**FIGURE 1:**
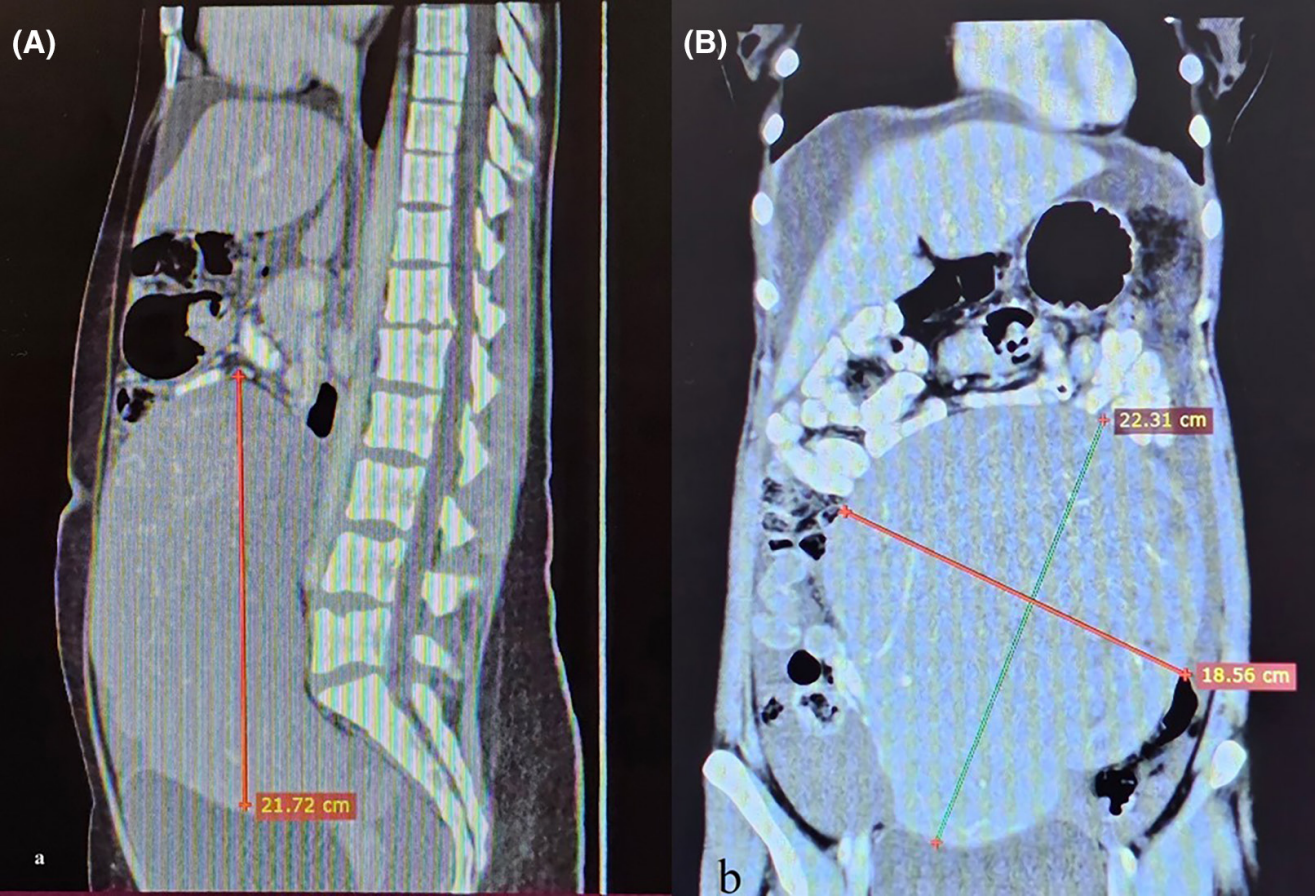
(A) Sagittal and (B) coronal computed tomography images showing intra‐abdominal mass measuring 22 cm (red arrow). Note the upward placement of other intra‐abdominal organs.

Ultrasonography revealed a solid right adnexal mass with a diameter of 20 cm, accompanied by mild hydronephrosis on the right side. The left ovary and uterus appeared normal. Subsequent computed tomography revealed a pelvic mass with a diameter of 22 cm, characterized by an indistinct border between the uterus and pelvic sidewalls. There was also evidence of right ureteral displacement (Figure 1A,B). Laboratory tests measuring alpha‐fetoprotein, carcinoembryonic antigen, and cancer antigen 125 (CA125) levels did not reveal any abnormal results.

**FIGURE 2:**
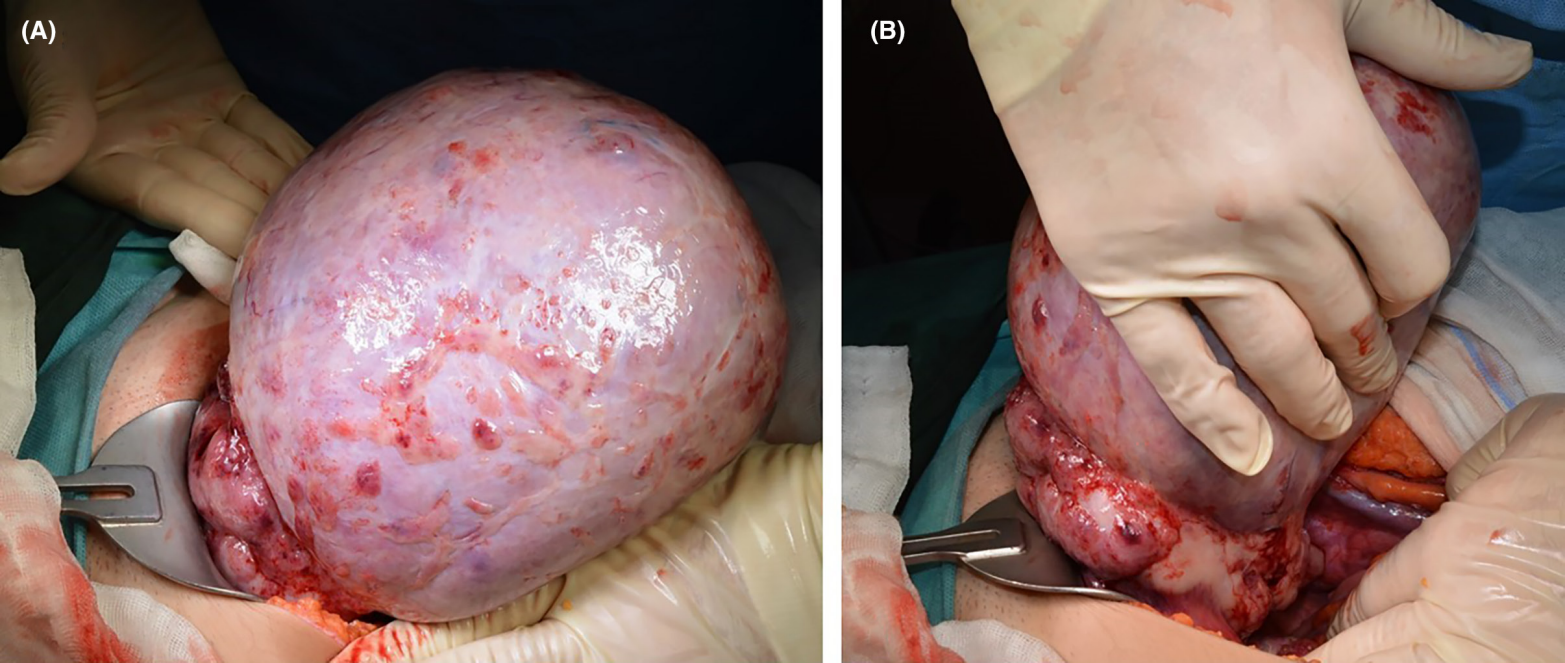
Laparotomy showed (A) the mass arising from (B) the right ovary.

Following the diagnosis of a right ovarian mass, the patient underwent surgical exploration via laparotomy. Consequently, a 22 cm right ovarian mass with external projections, moderate ascites, and peritoneal implants were observed intraoperatively (Figure 2A,B). The remainder of the abdominal cavity appeared normal, and ascitic fluid was sampled for cytological examination. Intraoperative cytological frozen analysis of the tumor revealed malignant cells of the germinal nature. Right salpingo‐oophorectomy was performed for mass excision, and a biopsy was taken from the left ovary, which appeared more prominent than average. Excision of all tumoral implants, pelvic and para‐aortic lymph node dissection, and omentectomy were also carried out. The patient received a total of 11 units of transfused erythrocytes, eight intraoperatively and three postoperatively, with no postoperative complications. She was discharged on the fifth day after the surgery.

### 2.3 Conclusion and results

**FIGURE 3:**
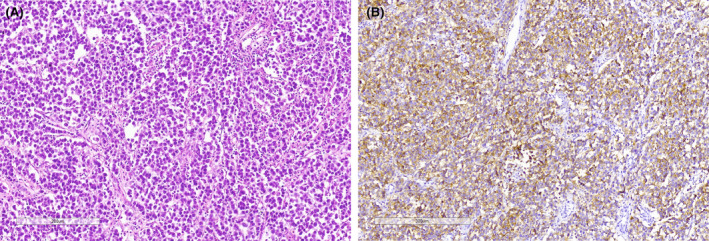
(A) Histological slide showing islands of uniform polygonal cells with clear or eosinophilic cytoplasm and distinct cytoplasmic membranes, surrounded by collagenous strands infiltrated by lymhocytes (Hematoxylin & Eosin, 20× magnification). (B) Immunohistochemistry for PLAP marks the tumor cells in a membranous and cytoplasmic fashion (Immunoperoxidase stain, 10× magnification).

The final histopathological examination revealed a pure dysgerminoma with multiple lymph node involvement and omental and peritoneal metastases (Figure 3A,B). Accordingly, she was diagnosed with the International Federation of Obstetrics and Gynecology (FIGO) 3AMOGCT, indicating that the cancer has spread to the serosa of the uterus and/or the tissue of the fallopian tubes and ovaries but not to other parts of the body. Subsequently, BEP (Bleomycin 30 units per week, Etoposide 100 mg/m^2^/day and cisplatin 20 mg/m^2^/day daily for Days 1–5) chemotherapy was administered at 3‐week intervals, starting from the 3rd postoperative week. She provided a complete clinical and radiological response after four cycles of BEP. She was followed up semi‐annually during the first year and then annually for the next 5 years with no signs of recurrence.

The initial examinations, including hysterosalpingography, which were performed due to the patient's desire to have children, indicated the normal functioning of the left salpinx. She had regular menstrual cycles occurring every 30 days and lasting 4–5 days. Nevertheless, she underwent in‐vitro fertilization since she could not get pregnant. The first attempt yielded only one mature oocyte with no fertilization. The second attempt yielded two oocytes, one successfully frozen at the blastocyst stage. However, before carrying out the transfer procedure, spontaneous pregnancy occurred. The patient experienced an uneventful pregnancy and gave birth to a healthy baby girl, during which ligation of the left uterine artery was performed to manage postpartum uterine atony.

## 3 DISCUSSION

The favorable fertility outcome in the case presented herein indicates that FSS with adjuvant BEP chemotherapy may be a reliable treatment alternative in patients with advanced dysgerminoma who desire to preserve their fertility. However, large‐scale studies are needed to validate the safety and feasibility of using this approach in advanced‐stage patients.

Post‐treatment pregnancy rates in patients who survived MOGCT are influenced by a range of sociodemographic and clinical factors, including age and desire for future motherhood.^5^ Solheim et al.^1^ reported an encouraging 87.2% post‐treatment pregnancy rate in patients who survived MOGCT attempting to get pregnant. Similarly, Chu et al.^2^ reported that 85.4% of the MOGCT patients with planned pregnancies had successful delivery. Tamauchi et al.^15^ reported that 40.0% of the 105 MOGCT patients who underwent FSS became pregnant after surgery, and 38.1% had successfully given birth, accounting for 95.2% of the patients who desired to become pregnant. On the other hand, there are also studies that reported relatively lower pregnancy and childbirth rates in this patient group.^5^, ^16^, ^17^

The discrepancies between reported pregnancy rates in this patient population may be due to the fact that all patients were taken into account in some studies, and only patients with pregnancy plans were taken into account when calculating the pregnancy rate in others. The lack of fertility evaluation in all patients included in the studies and the differences in follow‐up periods and evaluated number of pregnancies may also have contributed to the discrepancies between reported pregnancy rates in this patient population.^14^ In sum, independent risk factors predicting pregnancy outcomes remain controversial due to inconsistencies between relevant studies available in the literature. Large‐scale studies are needed to identify the independent risk factors that can predict pregnancy outcomes.

The number of cisplatin‐based chemotherapy cycles and cumulative doses of chemotherapeutics reportedly impact reproductive and sexual functions.^1^, ^18^ Several studies found a correlation between having three or fewer cisplatin‐based chemotherapy cycles and higher fertility rates.^1^, ^5^ In contrast, our patient achieved spontaneous pregnancy despite undergoing four cycles of chemotherapy. Similarly, Ghalleb et al.^13^ reported three full‐term natural pregnancies following FSS and six cycles of chemotherapy in MOGCT patients featuring a seminomatous component with an advanced‐stage yolk sac tumor.

FSS has been asserted as the primary treatment modality in patients with early‐stage (FIGO stages I and II) MOGCT.^5^, ^10^, ^15^, ^17^, ^19^ However, considering that most cases included in these studies were at an early stage, it can be argued that they could not accurately represent real‐world data. Husainiet et al.^16^ reported 32% as the pregnancy rate in patients with pure dysgerminoma, 33.8% of whom had FIGO stage III disease, indicating 87.5% of the patients who have been trying to get pregnant became pregnant. A study conducted in Iran^14^ reported the delivery rate as 73% in 26 patients who have been trying to become pregnant, approximately half of whom had FIGO stage III disease. The fact that our patient with FIGO stage III disease also gave a successful delivery supports the idea that many advanced‐stage MOGCT patients can achieve pregnancy after being treated with FSS coupled with adjuvant chemotherapy.

## 4 CONCLUSION

In the literature, FSS has been asserted as the primary treatment modality in patients with early‐stage MOGCT. In addition, age, desire to conceive, number of chemotherapy cycles, and cumulative doses of chemotherapeutics have been reported as major factors affecting pregnancy rates in patients who survive MOGCT. However, this case study demonstrated the potential for successful fertility preservation and pregnancy in an advanced‐stage MOGCT patient with pure dysgerminoma treated with FSS and adjuvant chemotherapy, indicating the potentially favorable outcomes of personalized treatment strategies in patients with advanced‐stage MOGCT.

In the literature, age, desire to conceive, number of chemotherapy cycles, and cumulative doses of chemotherapeutics have been reported as major factors affecting pregnancy rates in patients who survive MOGCT. Nevertheless, large‐scale studies are needed to verify the safety and feasibility of personalized treatment approaches in this patient population.

## AUTHOR CONTRIBUTIONS

**Göksu Göç:** Conceptualization; writing – original draft; writing – review and editing. **Alida Göç:** Investigation. **Gezim Kastrati:** Data curation. **Dardan Baftiu:** Visualization. **Fisnik Kurshumliu:** Resources; supervision.

## FUNDING INFORMATION

The authors received no financial support for the research or authorship of this article.

## CONFLICT OF INTEREST STATEMENT

The authors declare that they have no conflicts of interest.

## ETHICS STATEMENT

Informed consent was signed by the patient, and all identifiable data has been anonymized wherever possible in compliance with the Helsinki Declaration and local clinical research regulations (American Hospital Kosovo, Protocol # 416, September 2, 2022).

## CONSENT

Written informed consent was obtained from the patient to publish this report in accordance with the journal's patient consent policy.

## Data Availability

Data presented in this study are available from the corresponding author or first author on reasonable request.
